# Colonic Intussusception Secondary to Hereditary Angioedema

**DOI:** 10.14309/crj.0000000000000498

**Published:** 2021-01-13

**Authors:** Justin Roy, Rama Vunnam, Venkata Subhash Gorrepati, Deborah Bethards, Thomas McGarrity

**Affiliations:** 1 Department of Internal Medicine, Penn State Hershey Medical Center, Hershey, PA; 2 Division of Hospital Medicine, Penn State Hershey Medical Center, Hershey, PA; 3 Division of Gastroenterology and Hepatology, Penn State Health, Hershey, PA

## Abstract

Hereditary angioedema (HAE) is a rare genetic disease with numerous gastrointestinal manifestations. Intussusception, although rare, has been a reported complication with documentation of bowel wall edema on endoscopy during an acute flare. With the advent of synthetic C1 esterase inhibitors, this disease has become more effectively treatable. This case report shows a HAE flare complicated by colonic intussusception, treated with C1 esterase inhibitor, with complete endoscopic resolution seen on hospital day 5. This case provides evidence that with proper medical treatment, an HAE flare with intussusception has the potential to resolve without any further need for surgical or endoscopic intervention.

## INTRODUCTION

Patients with hereditary angioedema (HAE) usually present with gastrointestinal symptoms of nausea, vomiting, abdominal pain, and diarrhea. Although mostly self-limiting, rarely these symptoms might suggest underlying colonic intussusception. Advent of C1-inhibitor (C1-INH) therapy in 2012 changed the therapeutic landscape in treatment of symptoms with HAE. Here, we present such a case of young man with HAE who presented with colonic intussusception and was treated with C1-INH with complete resolution.

## CASE REPORT

A 27-year-old African American man with a medical history significant for HAE type 2 marked by a qualitative deficiency in endogenous C1-INH protein presented with abdominal pain that had worsened during the past 72 hours. He had been unable to obtain outpatient C1-INH therapy for the past 6 months because of financial constraints. He reported left lower quadrant, crampy pain with loose black stools during the past 2 weeks. He had a similar presentation a month before when computed tomography (CT) imaging showed intestinal edema from the gastric antrum to proximal duodenum. He was then treated with a dose of C1-INH but unfortunately left against medical advice.

**Figure 1.**
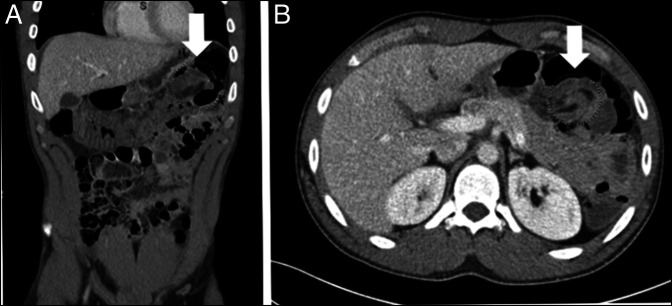
Abdominal and pelvic computed tomography with intravenous contrast demonstrating sequalae of hereditary angioedema including bowel edema and intussusception of the colon at the splenic flexure (non-obstructing) demonstrating (A) a coronal cut with intussusception marked by white arrow and (B) an axial cut with intussusception marked by white arrow.

On presentation, he was afebrile and hemodynamically stable. His examination showed a nondistended abdomen that was tender to palpation in the bilateral lower quadrants without rebound or guarding. Laboratory findings demonstrated a leukocytosis of 13.49k/μL, hemoglobin of 16.4 g/dL, and normal platelet count. The metabolic panel and liver chemistries were unremarkable. Abdominal and pelvic CT with contrast noted significant intestinal angioedema involving the bladder wall and transverse colon, most pronounced at the hepatic flexure, with telescoping of the large bowel at the splenic flexure consistent with nonobstructive intussusception, gastric, and small bowel edema seen on CT the previous month was not visualized (Figure 1).

**Figure 2.**
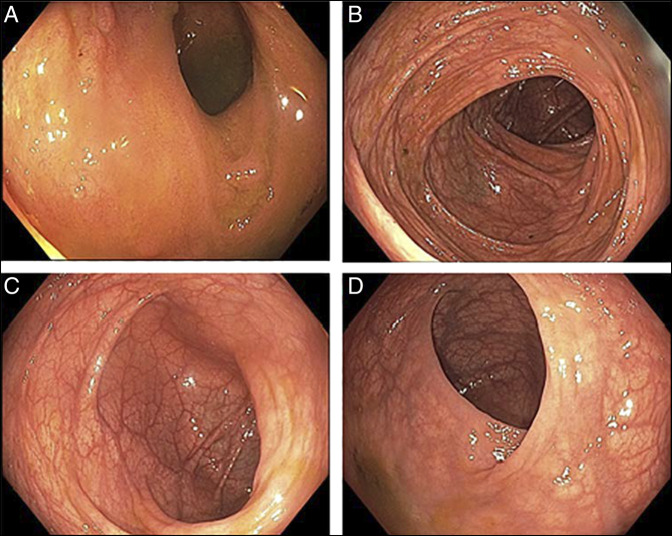
Colonoscopy on hospital day 5 after resolution of hereditary angioedema attack demonstrating normal appearing mucosa showing (A) terminal ileum, (B) transverse colon, (C) sigmoid colon, and (D) rectum.

He was promptly administered with C1-INH therapy at 20 mg/kg dosage. Because of ongoing symptoms, this dose was repeated twice in the subsequent 24 hours. Despite resolution of abdominal pain, the patient was noted to have ongoing dark stools during his hospitalization and underwent colonoscopy on day 5, which was unremarkable (Figure 2). The dark stools spontaneously resolved, and no clear etiology was identified. He was discharged with follow-up with immunology and did not require further hospitalization over the subsequent 90 days.

## DISCUSSION

HAE is a rare autosomal dominant disease that occurs in 3 major types—affecting the C1-INH protein quantitatively (type 1, 80%–85% of cases), qualitatively (type 2, 15%–20% of cases), and affecting coagulation factor XII (type 3, <5% of cases).^1^ This results in activation of the kallikrein-kinin system and release of vasoactive proteins causing the characteristic angioedema. It is estimated that HAE (all types) affects 1 in 10,000–50,000 persons across any ethnic group, although because of the rarity, the exact prevalence is unknown.^2^

The most common gastrointestinal manifestations include nausea and vomiting, colicky abdominal pain, and diarrhea, but intussusception in an adult is exceedingly rare (Table 1).^3–5^ Usually, flares or attacks of HAE are self-limited, lasting from 1 to 4 days before remitting.^6^ However, as described in the retrospective study above, 1 patient who presented with colonic intussusception required surgery.^3^ Interestingly, treatment with epinephrine, antihistamines, and corticosteroids are ineffective against HAE because they do not antagonize bradykinin.^7^ Hence, previous treatment included fresh frozen plasma and plasma kallikrein inhibitor.^8^ C1-INH proteins have been Food and Drug Administration approved for acute HAE episodes, and the intravenous medication we used on our patient (Berinert; CSL Behring GmbH, King of Prussia, PA) has been approved since 2012.^9,10^ Long-term prophylaxis is mainly achieved with plasma derived C1-INH, given subcutaneously at 60 IU per kg of body weight twice weekly or lanadelumab 300 mg subcutaneously every 2 weeks.

**Table 1.**
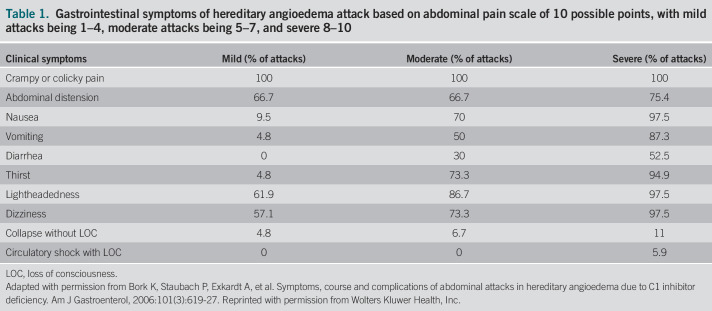
Gastrointestinal symptoms of hereditary angioedema attack based on abdominal pain scale of 10 possible points, with mild attacks being 1–4, moderate attacks being 5–7, and severe 8–10

Clinical symptoms	Mild (% of attacks)	Moderate (% of attacks)	Severe (% of attacks)
Crampy or colicky pain	100	100	100
Abdominal distension	66.7	66.7	75.4
Nausea	9.5	70	97.5
Vomiting	4.8	50	87.3
Diarrhea	0	30	52.5
Thirst	4.8	73.3	94.9
Lightheadedness	61.9	86.7	97.5
Dizziness	57.1	73.3	97.5
Collapse without LOC	4.8	6.7	11
Circulatory shock with LOC	0	0	5.9

LOC, loss of consciousness.

Adapted with permission from Bork K, Staubach P, Exkardt A, et al. Symptoms, course and complications of abdominal attacks in hereditary angioedema due to C1 inhibitor deficiency. Am J Gastroenterol, 2006:101(3):619-27. Reprinted with permission from Wolters Kluwer Health, Inc.

Colonic intussusception in adults is worrisome for malignancy because it occurs in 75% of such cases. Surgical adhesions may also be responsible; however, this patient had no previous surgical history. However, HAE is one of the rare benign causes for intussusception in adults.^11^ Before the development of C1-INH therapy, such patients underwent invasive procedures including air-contrast enemas and abdominal surgery.^3^ Now, treatment of the HAE flare with medical management and subsequent air enema is often sufficient to avoid surgical or endoscopic intervention.^11^

Koruth et al in 2005 described the endoscopic appearance of colonic HAE during an attack with the expected edematous bowel wall.^12^ To date, no published endoscopic imaging has been reported of an HAE-induced intussusception after treatment with C1-INH inhibitor. Although this case is limited by the fact that endoscopy was performed on day 5 (remembering that attacks spontaneously resolve in 1–4 days), it does show that complete endoscopic resolution is visible within 5 days of treatment. Furthermore, it marks another successful case of medical therapy resolving the intussusception without the need for invasive intervention.

## DISCLOSURES

Author contributions: J. Roy wrote the manuscript and is the article guarantor. R. Vunnam, GS Venkata, D. Bethards, and T. McGarrity edited the manuscript.

Financial disclosure: None to report.

Previous presentation: This case was accepted to the Society of General Internal National Meeting; May 6–9, 2020; Birmingham, Alabama.

Informed consent was obtained for this case report.
